# Poster Session I - A129 FERTILITY CLINIC REFERRAL RATES IN AN IBD PREGNANCY AND PRECONCEPTION CLINICAL RESEARCH COHORT

**DOI:** 10.1093/jcag/gwaf042.129

**Published:** 2026-02-13

**Authors:** J M Lee, H Li, V Srikanth, K O’ Connor, V Huang

**Affiliations:** Faculty of Arts and Sciences, University of Toronto, Toronto, ON, Canada; Faculty of Arts and Sciences, University of Toronto, Toronto, ON, Canada; Sinai Health, Toronto, ON, Canada; Sinai Health, Toronto, ON, Canada; Sinai Health, Toronto, ON, Canada

## Abstract

**Background:**

Inflammatory bowel disease (IBD), including Crohn’s Disease (CD) and ulcerative colitis (UC) often affects reproductive age women. In the general population, around 17% of North Americans experience infertility, and it is reported that only 10% of women seek fertility treatment. However, the literature surrounding fertility clinic referral rates of IBD patients and fertility attempt success rates in IBD patients are limited.

**Aims:**

This prospective cohort study aims to identify what percentage of mixed UC and CD IBD patients in cohort were referred to fertility clinics and to determine if CD or UC patients are referred to fertility clinics more.

**Methods:**

In this prospective cohort study, each pregnancy attempt from a patient in cohort in the Mount Sinai preconception and pregnancy in IBD clinical research program, was followed as an individual event and referred to as a journey. This prospective cohort study had 279 journeys total. Journeys that had confirmed fertility clinic referrals were separated from the rest of the cohort to form a study group. From this group, the percentage of IBD patients referred to fertility clinics was calculated. To study the differences between UC and CD patient’s referral rates, if a journey was not diagnosed with either CD or UC, it was removed from the study group. Journeys that had missing information regarding fertility treatment, or pregnancy outcomes were retrospectively charted. If the missing information was not available, those journeys were removed from the study group. Patients were then classified as either having a spontaneous conception before starting fertility treatment (NFT), fertility treatment resulting in conception (YFT), and fertility treatment with spontaneous conception (FTS).

**Results:**

Of the 279 journeys in cohort, 27 had fertility clinic referrals during preconception (∼9.7%). 1 journey was not a UC or CD patient and was removed from further analysis, leaving 26 journeys (13 UC and 13 CD patients). Out of the 26 journeys, 12 journeys were removed for insufficient data, leaving 14 (9 UC, 5 CD) journeys in the UC and CD patient study group. There were 7 NFT journeys, 4 YFT journeys, and 3 FTS journeys.

**Conclusions:**

About 9.7% of the total cohort population was referred to a fertility clinic, suggesting that the referral rate to fertility clinics for IBD patients is comparable to the general population (10%). With the removal of one non-IBD patient, 26 CD or UC patient journeys remained, with equivalent CD and UC patient referrals (13 UC, 13 CD), suggesting that neither are more likely to be referred for fertility support.

**Funding Agencies:**

Mount Sinai Health Department of Medicine Summer Studentship Award

